# Supplementary material for: Notochord vacuoles absorb compressive bone growth during zebrafish spine formation
Source: eLife. 2020 Jan 29;9:e51221. doi: 10.7554/eLife.51221 (PMC7012607; doi:10.7554/eLife.51221)
Supplement: Transparent reporting form [file elife-51221-transrepform.docx]

***eLife’s* transparent reporting form**

We encourage authors to provide detailed information *within their submission* to facilitate the interpretation and replication of experiments. Authors can upload supporting documentation to indicate the use of appropriate reporting guidelines for health-related research (see [EQUATOR Network](http://www.equator-network.org/%20)), life science research (see the [BioSharing Information Resource](https://biosharing.org/)), or the [ARRIVE guidelines](http://www.plosbiology.org/article/info:doi/10.1371/journal.pbio.1000412) for reporting work involving animal research. Where applicable, authors should refer to any relevant reporting standards documents in this form.

If you have any questions, please consult our Journal Policies and/or contact us: [editorial@elifesciences.org](mailto:editorial@elifesciences.org).

**Sample-size estimation**

- You should state whether an appropriate sample size was computed when the study was being designed
- You should state the statistical method of sample size computation and any required assumptions
- If no explicit power analysis was used, you should describe how you decided what sample (replicate) size (number) to use

Please outline where this information can be found within the submission (e.g., sections or figure legends), or explain why this information doesn’t apply to your submission:

Sample size was not determined a priori. The specific number of individuals that were imaged in each case are indicated in the figure legends. For all time course live imaging multiple fish (>3) were imaged for each developmental stage. Sample sizes were chosen based on variability and whether quantitative or qualitative assessments were needed. In long-term imaging experiments that require multiple imaging sessions sample number can be limited by survival of individuals. More variable phenomena/phenotype or quantitative assessments were analyzed with larger sample sizes. We followed the precedent set by Garcia and Bagwell et al. 2017 and Wopat et al. 2018.

When quantifying differences in body length we used a two way ANOVA to account for the two variables, size and age, in the analysis. For this experiment a minimum of 27 individuals were measured for each time point. For all cell volume, sphericity, cell counts, and notochord length measurements an unpaired t-test with welch’s correction was used. Welch’s removed the assumption that the standard deviations are the same for each group.

**Replicates**

- You should report how often each experiment was performed
- You should include a definition of biological versus technical replication
- The data obtained should be provided and sufficient information should be provided to indicate the number of independent biological and/or technical replicates
- If you encountered any outliers, you should describe how these were handled
- Criteria for exclusion/inclusion of data should be clearly stated
- High-throughput sequence data should be uploaded before submission, with a private link for reviewers provided (these are available from both GEO and ArrayExpress)

Please outline where this information can be found within the submission (e.g., sections or figure legends), or explain why this information doesn’t apply to your submission:

We define a biological replicate as a fish or image taken of a fish at the same developmental stage. Technical replicates would be repeating the experimental assay multiple times.

Fish length measurements: This experiment had a technical replicate of 1. However, at least 27 individuals were followed and measured for each case. Because we did not detect variability between clutches we decided detailed measurements in multiple replicates were unnecessary.

Image processing for bubble plots, notochord reconstructions, and cell measurements: For these experiments multiple fish were imaged at 48 hpf. For WT n=4. For spzl mutants n=5. Cell volume calculations were averaged between many cells from the biological replicates. For WT, n=559. For mutant, n=596. Statistics were done using an unpaired t-test with welch’s correction.

Sphericity over time: In this experiment the biological replicates were as follows; 48 hpf n=4, 9 dpf n=2, 12 dpf n=3, 16 dpf n=3, 20 dpf n=1, 24 dpf n=1. Only 1 biological replicate was used for the later time points due to image quality issues in older samples. Only high quality images were used. From here many technical replicates were measured for sphericity at each time point; 48 hpf n=559, 9 dpf n=271, 12 dpf n=386, 16 dpf n=327, 20 dpf n=116, 24 dpf n=57.

Mosaic expression of DN-GFP-rab32. The construct was injected and expressing fish were followed throughout spine development. This experiment was technically replicated >5 times with 4 biological replicates imaged for the final experiment reported in this manuscript.

Live imaging: In all cases of live imaging, there were at least 3 biological replicates at each developmental stage. This number increased significantly at critical time points during spine development. We repeated the imaging to get a detailed understanding of growth progression and vacuole dynamics at all developmental stages.

Light sheet imaging: This experiment was repeated twice for both wt and mutant fish. We found the same phenomenon in both replicates. Additionally, the reported results were corroborated in multiple imaging modalities.

Rescue experiments: This experiment was done with at least 6 technical replicates. Due to the variability and quality of injections (transient expression of DNA constructs is often toxic or causes injury of the embryo) this experiment required many repetitions to get enough biological replicates that could be definitively assessed and conclusions on vacuole size rescue could be drawn with confidence.

Sheath cell vacuolization: For this experiment hundreds of fish were injected with the dstyk construct. Of these, only fish that were expressing in the notochord sheath were imaged. Therefore, there were 12 biological replicates.

Nuclear distribution: This experiment was performed with a large number of biological replicates for the reported data. For both WT and mutant sections 15 fish were used for each experiment. From these 15 fish at least 20 sections were examined and nuclei distribution was plotted on a coordinate plane. Data was highly consistent across specimens.

Nuclear volume and sphericity: This experiment utilized the sections imaged in the above experiment. To determine nuclear size and sphericity many nuclei were measured. They were chosen based on the quality of the section as well as their placement in the middle of the section. No nuclei on the periphery of the section were used as to ensure the entire nuclei were reconstructed. For WT n=18 nuceli. For mutant n=16 nuclei. We reported 3 examples of each in the figure.

Spine kinking over time: We followed individual fish throughout spine development and measured the angle of spine kinking at all time points. This experiment was technically replicated 4 times, with a minimum of 12 biological replicates at the start of each experiment.

Notochord deformation and spine kinking index: The notochord deformation index assay was carried out with 4 for WT and 5 mutant biological replicates. The spine kinking index was carried out with 18 biological replicates. Consistency across biological replicates was high.

We repeated micro-CT analysis three times. At least 5 biological replicates were imaged in the cases reported in this manuscript.

RA experiment: This experiment was performed twice in triplicate, giving us a technical replicate of 6 for each condition. 10 fish were raised in each replicate which gives us a biological replicate of 60 fish for each condition. Many of the replicates perished prior to imaging and some were too sick to use as a reliable replicate after the week long RA treatment. For the sizes reported in the paper there was a biological replicate of 3 individuals imaged for each condition.

*nob* experiment: This experiment was performed twice. Biological replicates for WT were n=11 and for nob mutants n=16. In the case of the late stage (9.5 mm) cryosections, 2 fish were sectioned and stained for both WT and *nob* mutants.

**Statistical reporting**

- Statistical analysis methods should be described and justified
- Raw data should be presented in figures whenever informative to do so (typically when N per group is less than 10)
- For each experiment, you should identify the statistical tests used, exact values of N, definitions of center, methods of multiple test correction, and dispersion and precision measures (e.g., mean, median, SD, SEM, confidence intervals; and, for the major substantive results, a measure of effect size (e.g., Pearson's r, Cohen's d)
- Report exact p-values wherever possible alongside the summary statistics and 95% confidence intervals. These should be reported for all key questions and not only when the p-value is less than 0.05.

Please outline where this information can be found within the submission (e.g., sections or figure legends), or explain why this information doesn’t apply to your submission:

We used a two-way ANOVA with Sidak’s test to determine the p value for the length measurement experiment in figure 1B. There were two variables, length and time. Our n value was a minimum of 27 for all time points measured.

We used an unpaired t-test with welch’s correction for experiments in figure 1I,K,J,K. Error bars are standard deviation. *p* values are reported on each graph. N values for all cases are reported in the description above.

We used an unpaired t-test with welch’s correction for experiments in figure 8D,G. Error bars are standard deviation and *p* values are reported on each graph. N values are listed above.

Error bars for figure 9C are standard deviation. N values for all time points were 13 for mutant and 3 for WT.

Error bars for figure 69 are standard deviation. There was a n=4 for WT and n=5 for mutant in this case.

A paired t-test was used for sup figure 1C. *p* values and n values are listed in the figure legend.

(For large datasets, or papers with a very large number of statistical tests, you may upload a single table file with tests, Ns, etc., with reference to sections in the manuscript.)

**Group allocation**

- Indicate how samples were allocated into experimental groups (in the case of clinical studies, please specify allocation to treatment method); if randomization was used, please also state if restricted randomization was applied
- Indicate if masking was used during group allocation, data collection and/or data analysis

Please outline where this information can be found within the submission (e.g., sections or figure legends), or explain why this information doesn’t apply to your submission:

Samples were allocated into experimental groups based on developmental stages. In all WT cases standard length is used to group individuals. Due to the shortened axis in our mutant samples we could not use standard length to stage them. Therefore, we used days post-fertilization in conjunction with other biological staging indicators (presence of bone, development of the cranium, size of head) to properly group replicates.

Beyond confirming similar developmental stages fish were chosen at random for imaging.

**Additional data files (“source data”)**

- We encourage you to upload relevant additional data files, such as numerical data that are represented as a graph in a figure, or as a summary table
- Where provided, these should be in the most useful format, and they can be uploaded as “Source data” files linked to a main figure or table
- Include model definition files including the full list of parameters used
- Include code used for data analysis (e.g., R, MatLab)
- Avoid stating that data files are “available upon request”

Please indicate the figures or tables for which source data files have been provided:

All microscopy data has been archived and can be provided.

Light sheet movies are very large in size (~1GB each), but also can be provided

All micro-CT files are also very large but are ready to be uploaded if necessary.

An excel file with all the data for;

Figure 1b,1I,1J,1K,1L,1M,1N, figure 6E, figure 8D,8G, figure 9C, 9F, 9H, figure 10E, and sup. figure 1C.
